# Comparative study on the epidemiological characteristics and hazards of respiratory syncytial virus and influenza virus infections among elderly people

**DOI:** 10.1186/s12879-024-10048-1

**Published:** 2024-10-09

**Authors:** Jiangtao Yu, Na Liu, Yiheng Zhu, Wenyu Wang, Xianquan Fan, Xuan Yuan, Juan Xu, Benfeng Zheng, Lin Luan

**Affiliations:** 1 Department of Epidemiology, School of Public Health, National Vaccine Innovation Platform, Nanjing Medical University, Nanjing, 210000 PR China; 2 Center for Immunization Planning, Chinese Center for Disease Control and Prevention, Beijing, 100050 China; 3 Department of immunization Program, Suzhou Center for Disease Control and Prevention, Suzhou, 215000 China; 4 Suzhou Municipal Health Commission, Suzhou, 215002 China

**Keywords:** The elderly, Hospitalization, Respiratory syncytial virus, Influenza virus, Acute respiratory infection

## Abstract

**Objective:**

To investigate the epidemiological characteristics and infections of respiratory syncytial virus (RSV) and influenza viruses in hospitalized elderly patients with respiratory tract infections in Suzhou City, China, and to compare the differences in clinical characteristics and economic burden associated with these two infections.

**Methods:**

In this prospective study, pathogenetic testing and clinical data for hospitalized patients aged 60 years and older with respiratory tract infections were collected in five hospitals through stratified cluster sampling from December 2023 to May 2024. Comparative study on epidemic characteristics, clinical features and costs of cases who infected RSV alone and influenza alone were conducted.

**Results:**

Among 1,894 cases included, the RSV positivity rate was 5.91% during the 2023–2024 winter-spring season, while the influenza positivity rate was 9.61%. RSV-B was the predominant subtype of RSV, and influenza A (primarily H3N2) was the dominant strain among the influenza-positive cases. Compared with cases infected influenza virus alone, those infected RSV alone had lower occurrence frequency of fever (18.8% vs. 35.7%, *P* = 0.004), higher occurrence frequency of complications of lower respiratory tract infections (70.8% vs. 54.8%, *P* = 0.011), higher direct medical costs ($996.2 vs. $841.1, *P* = 0.017) and total costs ($1019.7 vs. $888.1, *P* = 0.036). RSV single infection is more common in female cases (*P* = 0.007) and diabetic cases (*P* = 0.007) than influenza virus single infection.

**Conclusions:**

During the winter and spring months, RSV is the second most common pathogen after influenza virus among older adults hospitalized for respiratory infections in Suzhou, China. Patients infected RSV are more likely to develop complications with lower respiratory tract infections and have higher medical costs than the influenza. RSV infection in the elderly should be emphasized, especially in female patients and diabetic patients.

**Supplementary Information:**

The online version contains supplementary material available at 10.1186/s12879-024-10048-1.

## Introduction

 Acute respiratory infection (ARI) is a syndrome characterized by fever, cough, sore throat, and other symptoms of respiratory tract infection. Influenza virus and respiratory syncytial virus (RSV) are common pathogens [1, 2]. RSV is a pathogen that primarily affects the respiratory system, causing upper respiratory tract infection (URTI) and lower respiratory tract infection (LRTI). Among these, lower respiratory tract infections include pneumonia, bronchitis, and bronchiectasis [3]. Children, individuals aged 60 and older, and those with compromised immune systems are more susceptible to RSV lower respiratory infections, which can be severe and even fatal. However, RSV, known primarily for its high prevalence in infants and young children, has received less attention in the elderly [4, 5]. Its impact on the elderly has not been emphasized until recent years, although it is now increasingly recognized as an important pathogen causing severe respiratory disease in the elderly and those with underlying comorbidities [6]. 

Globally, the severity and disease burden of RSV in older adults can be comparable to that of influenza [7, 8]. However, epidemiologic data for the elderly population are very limited compared to the extensive RSV-related literature for infants and young children. Notably, misdiagnosed or unrecognized RSV infections may lead to an underestimation of the RSV burden due to the similarity of the clinical manifestations of RSV and influenza. Studies have noted that 10-31% of adult RSV patients require intensive care, 3-17% require mechanical ventilation, and mortality can be 6-8% [9]. In a population-based study examining the proportion of acute respiratory illnesses caused by RSV and estimating the burden of disease in older adults, Nicholson et al. calculated that the incidence of wintertime respiratory illnesses caused by RSV exceeded that of nonpandemic influenza [10, 11]. These findings underscore the urgency of increased attention to RSV infection in older adults.

Therefore, this study conducted a 6-month pathogen surveillance of respiratory tract infections in the elderly in Suzhou City, China, to investigate the prevalence characteristics and proportion of RSV infections among the elderly, compare RSV and influenza virus infections in terms of clinical characteristics and disease burden, elucidate the hazards of RSV-associated respiratory infections on the health of the elderly population, and provide a basis for the development of RSV-associated preventive strategies.

## Materials and methods

### Surveillance and case definition

In this study, we collected clinical data from elderly patients aged 60 years and older who were hospitalized due to ARI at five hospitals in Suzhou City (Suzhou Ninth People’s Hospital, The People’s Hospital of SND, Suzhou Changshu Xinzhuang People’s Hospital, Zhangjiagang Hospital of Traditional Chinese Medicine, and The First People’s Hospital of Kunshan) and conducted pathogenicity testing from December 1, 2023, to May 31, 2024. The WHO and European case definitions of ARI for RSV infection surveillance were used: acute onset (within 10 days) with symptoms of respiratory infection (at least one symptom: cough, sore throat, shortness of breath, or runny nose) [12, 13]. 

### Methods

#### Study design and data collection

**Fig. 1:**
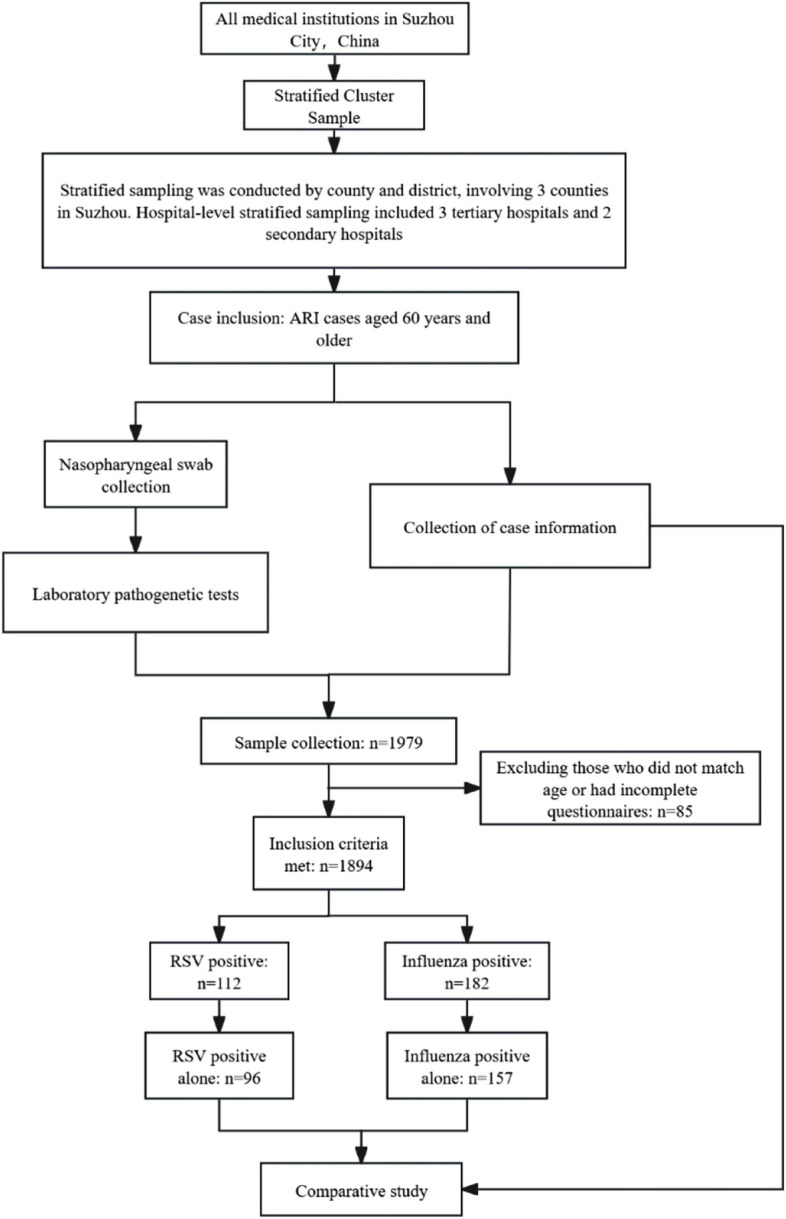
Flowchart of the study

This study was a prospective, multicenter study employing stratified whole cluster sampling. Stratified sampling was conducted by county and district, involving 3 counties in Suzhou (Zhangjiagang City, Changshu City, and Kunshan City) and 2 districts (Wujiang and Huqiu). Hospital-level stratified sampling included 3 tertiary hospitals: Suzhou Ninth People’s Hospital, Zhangjiagang Hospital of Traditional Chinese Medicine, and The First People’s Hospital of Kunshan; and 2 secondary hospitals: The People’s Hospital of SND and Suzhou Changshu Xinzhuang People’s Hospital. The sampled hospitals collected and sent samples for testing from all elderly patients hospitalized for acute respiratory infections. See Fig. 1.

The study used a self-designed survey form to collect case information, including: (1) demographic information (age, gender); (2) clinical information: clinical symptoms, underlying diseases, date of admission, date of discharge, discharge diagnosis, and hospitalization costs; (3) patient outcomes: including whether the patient died during the hospitalization period, time of death, whether they were admitted to an intensive care unit (ICU), and time of ICU admission; and (4) economic burden: including direct medical costs (comprehensive medical services, diagnosis, treatment, medication, and consumables), direct non-medical costs (transportation and escort costs), and indirect costs (lost labor costs due to patient and family accompaniment). These data were obtained through the hospital information system and patient questionnaires. The questionnaire was developed specifically for this study to assess the epidemiological characteristics, clinical characteristics and disease burden of RSV and influenza in older adults (see Supplementary Material 1 and Supplementary Material 2 for the English version).

#### Sample collection and laboratory tests

Respiratory samples (nasal swabs) were collected by local hospital professionals during the study participants’ visits to the clinic or hospitalization, following standard sampling procedures. The samples were placed in tubes containing viral sampling solution and stored in a refrigerator at 2–8℃ (for up to 48 h) or at -20℃ (for up to 1 week). Influenza Novel Coronavirus Respiratory 13 Pathogen Nucleic Acid Test Kit (Fluorescent PCR method) (Jiangsu Bioperfectus Technologies Co., Ltd.) and real-time fluorescent quantitative PCR were used to detect pathogens such as Respiratory syncytial virus, Influenza virus, Human adenovirus (HAdV), Human rhinovirus (HRV), Human parainfluenza virus (HPIV), Human coronavirus (HCoV, Not including COVID-19), Human metapneumovirus (HMPV), Chlamydia pneumoniae (CP), Mycoplasma pneumoniae, Streptococcus pneumoniae (SP), Human bocavirus (HBoV), Enterovirus (EV), and other pathogens. RSV-positive specimens were subtyped using the Duplex Real-Time PCR Diagnostic Kit for Rapid Detection of respiratory syncytial viruses A/B (Beijing Zhuocheng Huisheng Biotechnology Co.). Similarly, Influenza A-positive specimens were subtyped using the Duplex Real-Time PCR Diagnostic Kit for Rapid Subtyping of Human Influenza A Viruses (Beijing Zhuocheng Huisheng Biotechnology Co.).

#### Statistical methods

Excel 2019 software was used to summarize the data, and R 4.3.1 software was applied to process and analyze the data. The study subjects were divided into RSV single and influenza single infection groups for comparison. Continuous variables are presented in mean and standard deviation (SD) and compared by independent-samples t-test. Counting variables are presented in medians and interquartile ranges (IQR) and analyzed by Nonparametric test. The chi-square (*χ*^2^) test or Fisher’s exact test was used for count data. A P-value of < 0.05 was considered statistically significant.

## Results

### Pathogen infections of respiratory tract infections in the elderly

#### RSV and influenza infections in the elderly

**Fig. 2:**
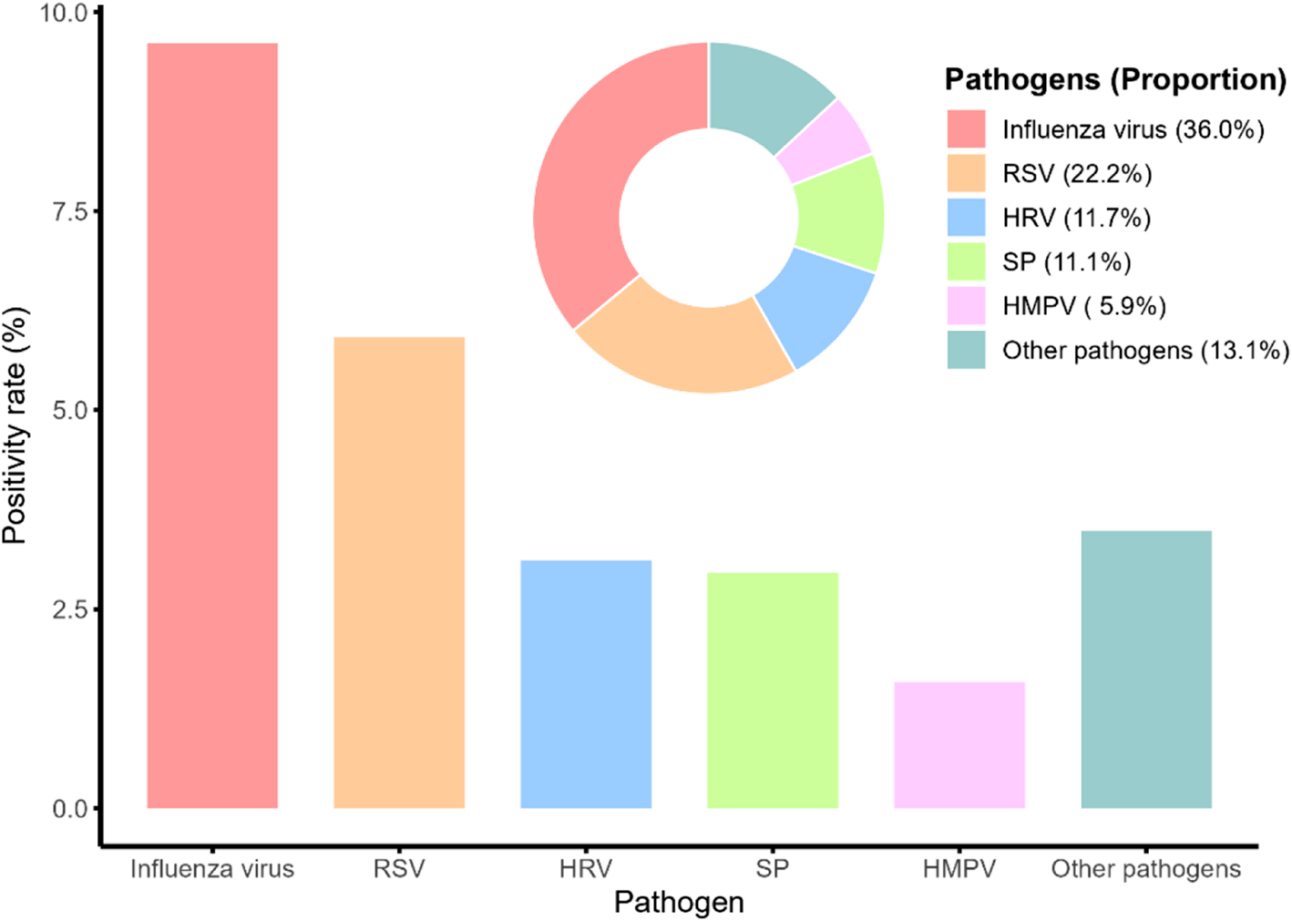
Proportion of pathogenic infections and positive detection of common pathogens of respiratory tract infections among the elderly in Suzhou City, China

From December 1, 2023, to May 31, 2024, a total of 1,894 cases of respiratory infections were investigated among the elderly in Suzhou City, with 455 testing positive for pathogens, yielding a positivity rate of 24.0%. Of these, 409 were infected with one pathogen, 42 with two, and four with three. The five pathogens with the highest detection rates were influenza virus (9.61%, 182/1,894), RSV (5.91%, 112/1,894), HRV (3.12%, 59/1,894), SP (2.96%, 56/1,894), and HMVP (1.58%, 30/1,894) (see Fig. 2). Influenza virus accounted for the highest percentage of positive pathogens (36.0%, 182/505), followed by RSV (22.2%, 112/505), HRV (11.7%, 59/505), SP (11.1%, 56/505), and HMVP (5.9%, 30/505) (see Fig. 2).

**Fig. 3:**
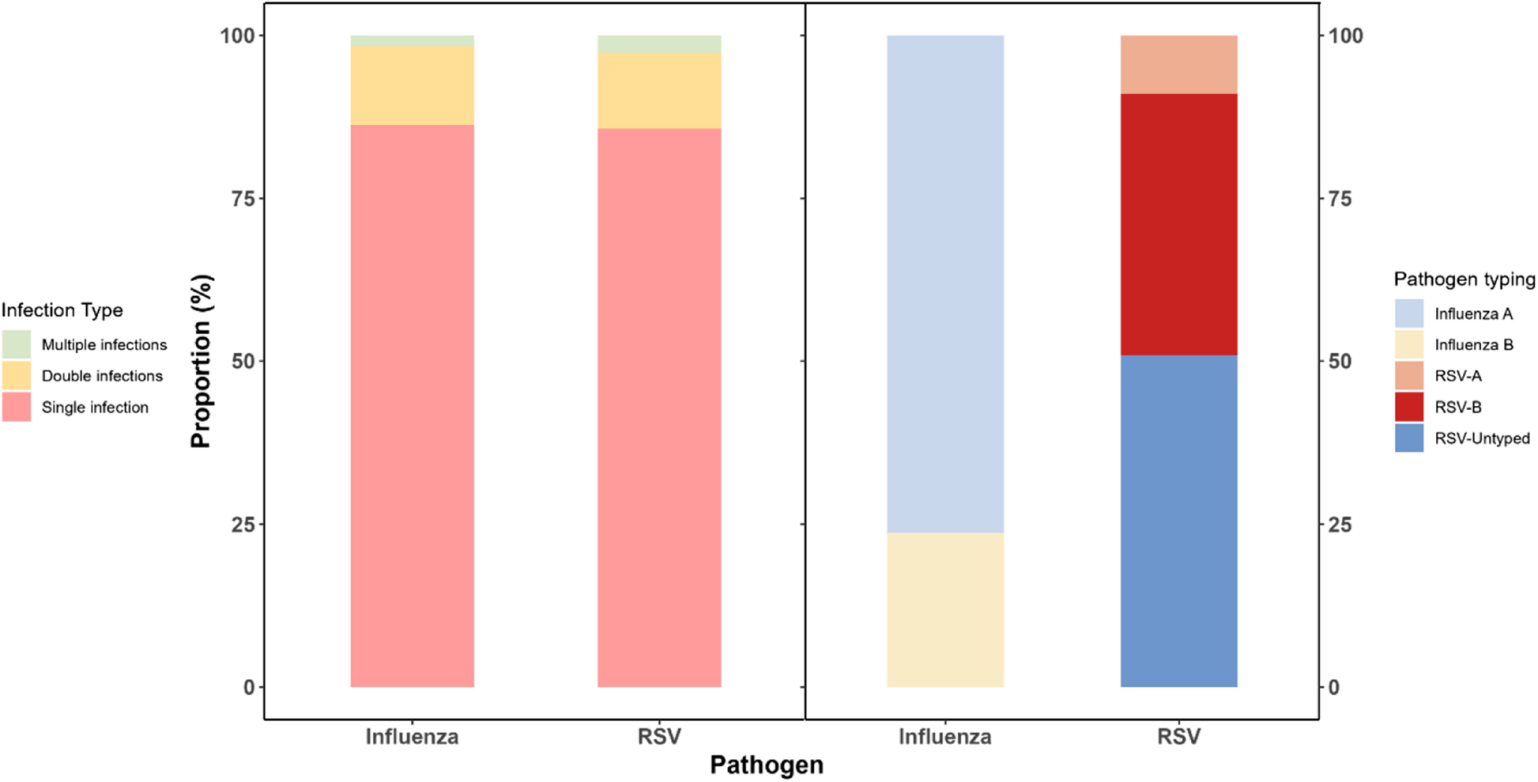
Types of Influenza and RSV infection, and pathogen typing in elderly people in Suzhou City, China

Among influenza virus infections, single infection accounted for 86.3% (157/182), double infections for 12.1% (22/182), and multiple infections for 1.6% (3/182). For RSV infections, single infection comprised 85.7% (96/112), double infections 11.6% (13/112), and multiple infections 2.7% (3/112) (see Fig. 3). Among influenza virus infections, influenza A accounted for 76.4% (139/182) and influenza B for 23.6% (43/182). Of the influenza A cases, 9.4% (*n* = 13) were H1N1, and 88.5% (*n* = 123) were H3N2, with no subtyping data available for 3 isolates. For RSV infections, subtypes were identified in 49.1% of patients (55/112). Among those with identified subtypes, RSV-A accounted for 18.2% (10/55) and RSV-B for 81.8% (45/55) (see Fig. 3).

#### RSV and influenza epidemic characteristics

**Fig. 4:**
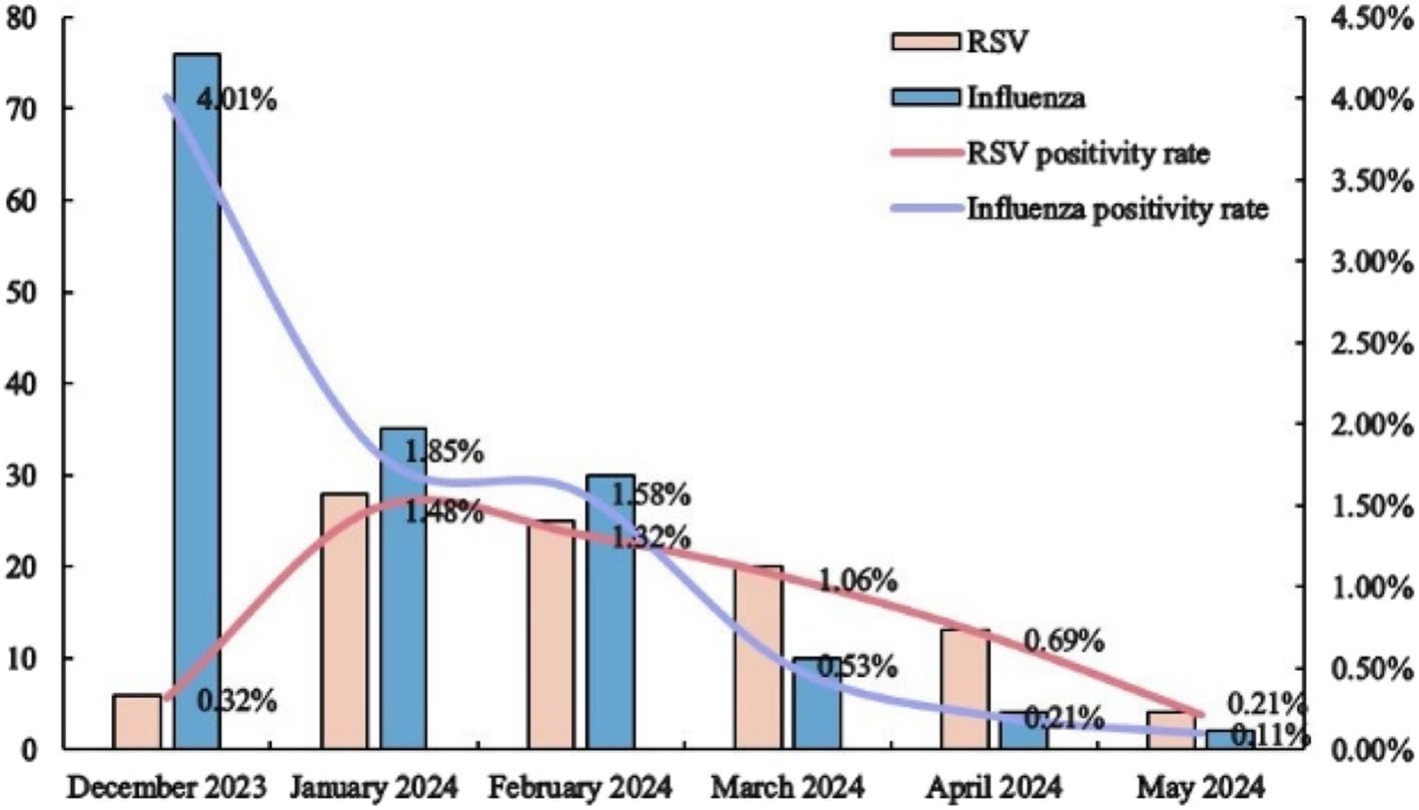
Distribution of the time of onset of RSV and influenza virus-associated respiratory infections in older adults

Influenza positivity was highest in December and then showed a significant month-to-month decline (*χ*^2^_trend_ = 101.27, *P* < 0.05). RSV positivity was significantly lower than influenza in December, but spiked to a peak in January and then declined month-to-month (*χ*^2^_trend_ = 9.84, *P* < 0.05). Overall, influenza positivity rates were higher than RSV positivity rates from December to February, but from March to May, RSV positivity rates were higher than influenza positivity rates (see Fig. 4).

#### Comparison of the basic information of RSV single and influenza virus single infection cases

The patients in both groups were mainly concentrated in the age group of 70–79 years. The mean age of those infected with RSV alone was 75.4 ± 7.6 years, while that of those infected with influenza virus alone was 75.7 ± 8.1 years.

RSV infections were 43.8% (42/96) in males and 56.2% (64/96) in females; influenza infections were 61.1% (96/157) in males and 38.9% (61/157) in females. The proportion of females infected with RSV was greater than that of influenza, and the difference was statistically significant (*P* = 0.007). RSV infections were more prevalent in the county-level city than influenza (69.8% vs. 56.7%), and the difference was statistically significant (*P* = 0.038).

Among the underlying diseases, 20.8% (20/96) of RSV-infected and 8.9% (14/157) of influenza-infected patients had diabetes, a difference that was statistically significant (*P* = 0.007). The difference between the two groups was not statistically significant for cardiovascular disease (62.5% vs. 57.3%), chronic respiratory disease (50.0% vs. 40.8%), and cancer (6.3% vs. 1.3%) (see Table 1).

**Table 1:** Basic characteristics of cases of respiratory syncytial virus and influenza virus-associated respiratory infections in the elderly [n (%)]

Categories	RSV single infection [*n* (%)]	Influenza virus single infection [*n* (%)]	χ^2^	*P*-value
**Sex**				
Male	42(43.8)	96(61.1)	7.272	0.007
Female	54(56.2)	61(38.9)		
**Age**				
60–69	20(20.8)	38(24.2)	0.388	0.850
70–79	47(49.0)	73(46.5)		
≥ 80	29(30.2)	46(29.3)		
**Region of residence**				
Urban	29(30.2)	68(43.3)	4.327	0.038
County-level city	67(69.8)	89(56.7)		
**Occupation**				
Farmer	54(56.3)	103(65.6)	-	0.238*
Worker	2(2.1)	5(3.2)		
Retired staff	40(41.7)	49(31.2)		
**Underlying disease**				
Cardiovascular disease	60(62.5)	90(57.3)	0.661	0.416
Diabetes	20(20.8)	14(8.9)	7.272	0.007
Chronic respiratory diseases	48(50.0)	64(40.8)	2.060	0.151
Cancer	6(6.3)	2(1.3)	-	0.056*

*Using Fisher's exact probability method

#### Comparison of the clinical features of RSV single and influenza virus single infection cases

The most common symptoms of RSV infection in elderly patients infected with RSV alone at the time of admission were cough (96.9%), followed by sputum production (62.5%), dyspnea (29.2%), fever (18.8%), shortness of breath (18.8%), wheezing (15.6%), runny nose (11.5%), and sore throat (10.4%). The most common symptom of influenza virus infection was cough (94.3%), followed by sputum production (52.9%), fever (35.7%), dyspnea (24.2%), wheezing (21.7%), shortness of breath (14.6%), sore throat (14.0%), and runny nose (13.4%). The proportion of fever was lower in RSV-infected elderly patients (18.8%) compared to those with influenza virus infection (35.7%) (*P* = 0.003). RSV infection led to LRTI complications (pneumonia, bronchiolitis) in 70.8% (68/96) of cases, while influenza virus infection led to LRTI complications in 54.8% (86/157) of cases, and the difference was statistically significant (*P* = 0.011) (see Table 2).

**Table 2:** Comparison of clinical characteristics of hospitalized cases of respiratory syncytial virus and influenza virus-associated respiratory infections in the elderly [n (%)]

Categories	RSV single infection [n (%)]	Influenza virus single infection [n (%)]	t/χ^2^	*P*-value
**Symptom**				
Fever	18(18.8)	56(35.7)	8.240	0.004
Cough	93(96.9)	148(94.3)	-	0.544*
Runny nose	11(11.5)	21(13.4)	0.198	0.656
Sputum production	60(62.5)	83(52.9)	2.250	0.134
Sore throat	10(10.4)	22(14.0)	0.697	0.404
Wheezing	15(15.6)	34(21.7)	1.388	0.239
Shortness of breath	18(18.8)	23(14.6)	0.738	0.390
Dyspnea	28(29.2)	38(24.2)	0.761	0.383
**Complications of LRTI (pneumonia**,** bronchitis)**	68(70.8)	86(54.8)	6.448	0.011
**Oxygen saturation (%)**				
< 90	3(3.1)	9(5.7)	-	0.544*
**ICU admissions**				
Yes	1(1.0)	6(3.8)	-	0.258*
**Days of hospitalization(Mean ± SD)**	11.0 ± 3.7	11.2 ± 4.3	-0.417	0.677
**Discharge outcomes**				
Recovered	6(6.3)	12(7.6)	-	0.594*
Partially healed	89(92.7)	140(89.2)		
Not healed	1(1.0)	5(3.2)		
Death	0(0)	0(0)		

*Abbreviation*: *SD* Standard deviation

*Using Fisher's exact probability method

There was no statistically significant difference between RSV-infected cases and those with influenza virus infection in terms of oxygen saturation, ICU admissions, days of hospitalization, and discharge outcomes (see Table 2).

#### Comparison of the economic burden of disease in RSV single and influenza virus single infection cases

The direct medical costs were $996.2 (792.0-1324.7) for RSV-infected cases and $841.1(574.5-1335.9) for influenza virus-infected cases, which was statistically higher for RSV infection than for influenza virus infection (*P* = 0.017). The difference in direct non-medical costs and indirect costs of RSV infection compared to influenza virus infection was not statistically significant (see Table 3).

**Table 3:** Comparison of hospitalization costs for hospitalized cases of respiratory syncytial virus and influenza virus-associated respiratory infections in the elderly [M(IQR), $]

Type of costs	RSV single infection [Median (IQR)]	Influenza virus single infection [Median (IQR)]	Z	*P*-value
Direct medical costs	996.2(792.0-1324.7)	841.1(574.5-1335.9)	-2.395	0.017
Direct non-medical costs	0(0-5.2)	1.4(0-6.9)	-1.389	0.165
Indirect costs	0(0–0)	0(0–47.0)	-1.523	0.128
Total costs	1019.7(808.6-1432.2)	888.1(601.5-1412.7)	-2.092	0.036

*Abbreviation:* *IQR* Interquartile range

The total economic burden of RSV infection was higher than influenza virus infection ($1019.7 vs. $888.1), and the difference was statistically significant (*P* = 0.036) (see Table 3).

## Discussion

During the winter and spring seasons of 2023–2024, respiratory infections among older adults in Suzhou City, China, were predominantly caused by single-pathogen infections, with influenza virus being the most common. Influenza A was the main pathogen, with H3N2 identified as the dominant subtype. This aligns with findings by André Almeida et al., who reported H3N2 as the predominant influenza A subtype in elderly populations [14]. Similarly, a study by Sara Carazo et al. found that influenza A (H3N2) was associated with a higher risk of hospitalization compared to influenza A (H1N1) and influenza B [15]. Therefore, subtype surveillance is crucial for monitoring influenza infections in the elderly. After the COVID-19 pandemic onset and the introduction of non-pharmaceutical interventions (NPIs) there was evidence of the absolute predominance of RSV-A [16]. However, in the present study, RSV-B emerged as the dominant subtype, potentially signaling a return to the previous pattern of co-circulation of RSV-A and RSV-B.

Since the onset of the COVID-19 pandemic and the implementation of corresponding NPIs, the rate of respiratory pathogen infections has been significantly impacted [17]. RSV has been found to be more susceptible to NPIs than influenza viruses [17]. One study showed that the incidence of RSV infections was significantly lower during the COVID-19 pandemic compared to the pre-pandemic period [18]. The RSV positivity rate among older adults in Beijing, China, decreased significantly during the COVID-19 pandemic, reaching its lowest level in 9 years [19]. In Shenzhen, China, the RSV positivity rate in January 2023 was even 0% [20]. However, after the end of the COVID-19 pandemic, RSV positivity among older adults in Beijing exceeded both pre-pandemic and pandemic levels, peaking in December 2023 at its highest level in 9 years [19]. This study also found that the RSV positivity rate in the Suzhou area peaked in January 2024 and lasted longer compared to influenza. Although both positivity rates declined after the winter peak, the decrease in RSV was more gradual, and its positivity rate exceeded that of influenza from March to May. These findings suggest that following the COVID-19 pandemic and the lifting of NPIs, RSV has resumed its typical transmission pattern.

This study of respiratory pathogen surveillance in older adults over a 6-month period suggests that the health risks of RSV infection in this population cannot be ignored. Results from a population-wide ARI prospective surveillance in China, conducted from 2009 to 2019, showed that RSV ranked 5th among the 8 pathogens monitored, with a det ection rate of 7.4% [21]. Among the pathogens monitored in our study, the detection percentage of RSV ranked 2nd, only behind influenza, and was higher than in the 2009–2019 study. This suggests that RSV constitutes a significant proportion of respiratory infections in elderly individuals in Suzhou and is one of the most important pathogens causing respiratory infections in this population.

Clinical symptom of RSV infection are similar to those of other viral respiratory pathogens and include cough (more than 90%), nasal congestion and runny nose (22-78%), sore throat (16-64%), and dyspnea (51-93%) [22]. Differentiating RSV infection from influenza virus infection can be challenging without laboratory pathogenetic tests. Although there is significant symptom overlap, studies have found that influenza virus infections are more likely to present with fever (temperature ≥ 38℃) [23], whereas RSV infections are often non-febrile or present with only a low-grade fever [24]. Additionally, RSV infections are more commonly associated with symptoms of runny nose and wheezing compared to influenza, while fever over 38℃ is more frequently observed in influenza infections [13, 25–27]. This study did not find any significant difference between RSV and influenza in terms of symptoms such as runny nose and wheezing. However, a notable difference was observed regarding fever. Fewer individuals with RSV infection experienced fever compared to those with influenza virus infection, and RSV infections more commonly presented with a low-grade fever. This characteristic may be useful for differentiating between RSV and influenza virus infections.

An interesting finding in this study is that the proportion of females infected with RSV is higher than that of females infected with influenza. A related study indicated that the risk of RSV infection in females may be greater compared to influenza [28]. This may be due to females spending more time with young children, who have a high prevalence of RSV, thereby indirectly increasing the risk of infection. Additionally, previous studies have shown that gender plays a key role in the incidence, immune response, and severity of ARI [29–31]. Therefore, the impact of RSV infection in females warrants particular attention.

The study reported by Hakan Sivgin et al. revealed that heart failure, hypertension, and diabetes were the underlying conditions associated with the highest risk of RSV hospitalization, with all severe cases in their study involving patients with diabetes [32]. Diabetes and hypertension are established risk factors for RSV, as reported in several prior studies [33–35]. Additionally, previous research has shown that adults hospitalized for RSV tend to be slightly older and have a higher prevalence of underlying medical conditions, particularly cardiopulmonary diseases, compared to those hospitalized for influenza virus infection [36–38]. Our study also found that a higher proportion of patients with diabetes were infected with RSV compared to those infected with influenza virus. Although no significant differences were observed between RSV and influenza in patients with hypertension and chronic respiratory disease, a higher proportion of patients hospitalized with RSV had hypertension (62.5% vs. 57.3%) and chronic respiratory disease (50.0% vs. 40.8%) compared to those hospitalized with influenza. Therefore, greater attention should be given to RSV patients with underlying conditions, especially those with diabetes.

Our study found that the length of hospitalization for RSV was similar to that for influenza, consistent with previous research [36, 37]. Despite similar hospitalization durations, the financial burden for older patients hospitalized with RSV was higher than for those with influenza. This finding is mentioned in a study by Bradley Ackerson et al., which reported that the medical costs for hospitalized RSV infected cases in individuals over 60 years of age were higher than those for influenza infected cases ($16,034 versus $15,163) [39]. RSV has long been recognized as a significant cause of severe illness in children, but many healthcare professionals may not fully appreciate that RSV is also a major pathogen responsible for the hospitalization of elderly patients with respiratory tract infections [22, 40, 41]. Both RSV and influenza virus infections exhibit seasonal epidemics during winter and spring, with overlapping time frames [42, 43]. The seasonal overlap, coupled with the infrequent use of RSV pathogen testing in clinical settings, likely contributes to the under-recognition of RSV infection risk among the elderly in previous studies. Unlike viruses for which effective vaccines and specific antiviral medicine are available, such as the influenza virus [44], there are currently no specific and effective treatments or interventions for RSV infection in China. This may partly explain why the disease burden of hospitalized elderly patients with RSV infection was higher than that of influenza virus infection in this study. Therefore, the significant disease burden of RSV infection in the elderly should not be overlooked.

RSV can cause severe lower respiratory tract infections in the elderly, potentially leading to respiratory failure and high mortality. Studies have shown that over 70% of RSV patients experience severe lower respiratory complications, including pneumonia and acute bronchitis [45, 46]. Our study also found that 70.8% of RSV-infected patients developed LRTI complications. An observational, retrospective cohort study by Ackerson et al. compared mortality and morbidity among adults aged ≥ 60 years hospitalized for RSV infection (*n* = 645) versus influenza (*n* = 1,878). The study found that RSV-infected individuals had a 2.7-fold higher risk of pneumonia (*P* < 0.001) compared to those with influenza, along with longer hospital stays and higher mortality rates [38]. A study by Ann R. Falsey et al. demonstrated that RSV positivity was more likely to result in hospitalization compared to other viral respiratory infections [13]. Similarly, Lee et al. found that hospitalized patients with RSV in Hong Kong were more likely to develop LRTI than those with influenza [37]. This finding is consistent with our study, which observed a higher proportion of LRTI complications among RSV-infected older adults (70.8%) compared to those with influenza (54.7%). Thus, RSV infection in the elderly may lead to more severe disease outcomes, which helps explain the higher economic burden of RSV compared to influenza in this study.

This study has several limitations. First, monitoring was conducted over a six-month period, and a full year of data collection has yet to be completed. Second, the study population included only individuals aged 60 years and older, excluding those under 60 years. We will continue monitoring to achieve a full year of surveillance and plan to conduct population-wide RSV surveillance in the future.

## Conclusions

The RSV epidemic in older adults in Suzhou City, China, occurs around the similar time as influenza and accounts for a significant proportion of respiratory infections in this population, with an economic burden comparable to that of influenza virus infection. RSV infections are more likely to lead to more serious lower respiratory complications than influenza infections. Therefore, establishing an RSV surveillance system should be prioritized to better monitor RSV infection in the elderly, particularly those with underlying conditions.

## Supplementary Information

Supplementary Material 1.

Supplementary Material 2.

Supplementary Material 3.

## Data Availability

The datasets generated and/or analyzed during the current study are not publicly available because of ethical and legal reasons but are available from the corresponding author on reasonable request.

## Abbreviations

RSV: Respiratory syncytial virus
ARI: Acute respiratory infection
URTI: Upper respiratory tract infection
LRTI: Lower respiratory tract infection
HAdV: Human adenovirus
HRV: Human rhinovirus
HPIV: Human parainfluenza virus
HCoV: Human coronavirus
HMPV: Human metapneumovirus
CP: Chlamydia pneumoniae
SP: Streptococcus pneumoniae
HBoV: Human bocavirus
EV: Enterovirus
NPIs: non-pharmaceutical interventions
SD: standard deviation
IQR: interquartile range

## References

[1] Tian J, Liu C, Wang X, Zhang L, Zhong G, Huang G, et al. Comparative analysis of clinical features of lower respiratory tract infection with respiratory syncytial virus and influenza virus in adults: a retrospective study. BMC Pulm Med. 2023;23:350. 37715219 10.1186/s12890-023-02648-5 PMC10504734

[2] Chen G, Lan M, Lin S, Zhang Y, Zhang D, Weng Y, et al. Genome analysis of human respiratory syncytial virus in Fujian Province, Southeast China. Infect Genet Evol J Mol Epidemiol Evol Genet Infect Dis. 2022;103:105329. 10.1016/j.meegid.2022.105329 35788050

[3] Lee CYF, Khan SJ, Vishal F, Alam S, Murtaza SF. Respiratory syncytial virus prevention: a new era of vaccines. Cureus. 2023;15:e45012. 37829940 10.7759/cureus.45012 PMC10565597

[4] Zhang N, Wang L, Deng X, Liang R, Su M, He C, et al. Recent advances in the detection of respiratory virus infection in humans. J Med Virol. 2020;92:408–17. 31944312 10.1002/jmv.25674 PMC7166954

[5] Nam HH, Ison MG. Respiratory syncytial virus infection in adults. BMJ. 2019;366:l5021. 31506273 10.1136/bmj.l5021

[6] Savic M, Penders Y, Shi T, Branche A, Pirçon J. Respiratory syncytial virus disease burden in adults aged 60 years and older in high-income countries: a systematic literature review and meta‐analysis. Influenza Other Respir Viruses. 2022;17:e13031. 36369772 10.1111/irv.13031 PMC9835463

[7] Díez-Domingo J, Pérez-Yarza EG, Melero JA, Sánchez-Luna M, Aguilar MD, Blasco AJ, et al. Social, economic, and health impact of the respiratory syncytial virus: a systematic search. BMC Infect Dis. 2014;14:544. 25358423 10.1186/s12879-014-0544-x PMC4219051

[8] Kestler M, Muñoz P, Mateos M, Adrados D, Bouza E. Respiratory syncytial virus burden among adults during flu season: an underestimated pathology. J Hosp Infect. 2018;100:463–8. 29614245 10.1016/j.jhin.2018.03.034

[9] Coussement J, Zuber B, Garrigues E, Gros A, Vandueren C, Epaillard N, et al. Characteristics and outcomes of patients in the ICU with respiratory syncytial virus compared with those with influenza infection: a multicenter matched cohort study. Chest. 2022;161:1475–84. 35063450 10.1016/j.chest.2021.12.670

[10] Nicholson KG, Kent J, Hammersley V, Cancio E. Acute viral infections of upper respiratory tract in elderly people living in the community: comparative, prospective, population based study of disease burden. BMJ. 1997;315:1060–4. 9366736 10.1136/bmj.315.7115.1060 PMC2127683

[11] Nicholson KG. Impact of influenza and respiratory syncytial virus on mortality in England and Wales from January 1975 to December 1990. Epidemiol Infect. 1996;116:51–63. 8626004 10.1017/s0950268800058957 PMC2271234

[12] Teirlinck AC, Broberg EK, Stuwitz Berg A, Campbell H, Reeves RM, Carnahan A, et al. Recommendations for respiratory syncytial virus surveillance at the national level. Eur Respir J. 2021;58:2003766. 33888523 10.1183/13993003.03766-2020 PMC8485062

[13] Falsey AR, McElhaney JE, Beran J, van Essen GA, Duval X, Esen M, et al. Respiratory syncytial virus and other respiratory viral infections in older adults with moderate to severe influenza-like illness. J Infect Dis. 2014;209:1873–81. 24482398 10.1093/infdis/jit839 PMC4038137

[14] Almeida A, Boattini M, Christaki E, Moreira Marques T, Moreira I, Cruz L, et al. Comparative virulence of seasonal viruses responsible for lower respiratory tract infections: a southern European multi-centre cohort study of hospital admissions. Infection. 2021;49:483–90. 33389699 10.1007/s15010-020-01569-3 PMC7778853

[15] Carazo S, Guay C-A, Skowronski DM, Amini R, Charest H, De Serres G, et al. Influenza hospitalization Burden by Subtype, Age, Comorbidity, and Vaccination Status: 2012–2013 to 2018–2019 Seasons, Quebec, Canada. Clin Infect Dis. 2024;78:765–74. 37819010 10.1093/cid/ciad627

[16] Eden J-S, Sikazwe C, Xie R, Deng Y-M, Sullivan SG, Michie A, et al. Off-season RSV epidemics in Australia after easing of COVID-19 restrictions. Nat Commun. 2022;13:2884. 35610217 10.1038/s41467-022-30485-3 PMC9130497

[17] Principi N, Autore G, Ramundo G, Esposito S. Epidemiology of respiratory infections during the COVID-19 pandemic. Viruses. 2023;15: 1160. 37243246 10.3390/v15051160 PMC10224029

[18] Juhn YJ, Wi C-I, Takahashi PY, Ryu E, King KS, Hickman JA, et al. Incidence of respiratory syncytial virus infection in older adults before and during the COVID-19 pandemic. JAMA Netw Open. 2023;6:e2250634. 36662530 10.1001/jamanetworkopen.2022.50634 PMC9860520

[19] Wei X, Li M, Wang Y, Huang Q, Gong C, Suo L, et al. Epidemiological and clinical characteristics of respiratory syncytial virus infection among people aged 60 and above in Beijing City. Chin J Prev Med. 2024;07:952–8. 10.3760/cma.j.cn112150-20240117-00065 39034779

[20] Sun Y, Wu W, Huang Y, Fang S, Liu H, Jiang M, et al. Epidemiological characteristics of human respiratory syncytial virus in influenza-like illness in Shenzhen City from 2019 to 2023. Chin J Prev Med. 2024;08:1117–23. 10.3760/cma.j.cn112150-20240318-00223 39142877

[21] Li Z-J, Zhang H-Y, Ren L-L, Lu Q-B, Ren X, Zhang C-H, et al. Etiological and epidemiological features of acute respiratory infections in China. Nat Commun. 2021;12:5026. 34408158 10.1038/s41467-021-25120-6 PMC8373954

[22] Branche AR, Falsey AR. Respiratory syncytial virus infection in older adults: an under-recognized problem. Drugs Aging. 2015;32:261–9. 25851217 10.1007/s40266-015-0258-9

[23] Call SA, Vollenweider MA, Hornung CA, Simel DL, McKinney WP. Does this patient have influenza? JAMA. 2005;293:987–97. 15728170 10.1001/jama.293.8.987

[24] Saha S, Pandey BG, Choudekar A, Krishnan A, Gerber SI, Rai SK, et al. Evaluation of case definitions for estimation of respiratory syncytial virus associated hospitalizations among children in a rural community of northern India. J Glob Health. 2015;5:010419. 26649172 10.7189/jogh.05.020419 PMC4652925

[25] Volling C, Hassan K, Mazzulli T, Green K, Al-Den A, Hunter P, et al. Respiratory syncytial virus infection-associated hospitalization in adults: a retrospective cohort study. BMC Infect Dis. 2014;14:665. 25494918 10.1186/s12879-014-0665-2 PMC4269936

[26] Widmer K, Griffin MR, Zhu Y, Williams JV, Talbot HK. Respiratory syncytial virus- and human metapneumovirus-associated emergency department and hospital burden in adults. Influenza Other Respir Viruses. 2014;8:347–52. 24512531 10.1111/irv.12234 PMC3984605

[27] Sundaram ME, Meece JK, Sifakis F, Gasser RA, Belongia EA. Medically attended respiratory syncytial virus infections in adults aged ≥ 50 years: clinical characteristics and outcomes. Clin Infect Dis off Publ Infect Dis Soc Am. 2014;58:342–9. 10.1093/cid/cit767 PMC7108027 24265361

[28] Begley KM, Monto AS, Lamerato LE, Malani AN, Lauring AS, Talbot HK, et al. Prevalence and clinical outcomes of respiratory syncytial virus vs influenza in adults hospitalized with Acute Respiratory Illness from a prospective Multicenter Study. Clin Infect Dis off Publ Infect Dis Soc Am. 2023;76:1980–8. 10.1093/cid/ciad031 PMC10250013 36694363

[29] Falagas ME, Mourtzoukou EG, Vardakas KZ. Sex differences in the incidence and severity of respiratory tract infections. Respir Med. 2007;101:1845–63. 17544265 10.1016/j.rmed.2007.04.011

[30] Klein SL. Sex influences immune responses to viruses, and efficacy of prophylaxis and treatments for viral diseases. BioEssays News Rev Mol Cell Dev Biol. 2012;34:1050–9. 10.1002/bies.201200099 PMC4120666 23012250

[31] Klein SL, Flanagan KL. Sex differences in immune responses. Nat Rev Immunol. 2016;16:626–38. 27546235 10.1038/nri.2016.90

[32] Sivgin H, Cetin S, Ulgen A, Li W. Diabetes and bacterial co-infection are two independent risk factors for respiratory syncytial virus disease severity. Front Med. 2023;10: 1231641. 10.3389/fmed.2023.1231641 PMC10646962 38020119

[33] Walsh EE, Peterson DR, Falsey AR. Risk factors for severe respiratory syncytial virus infection in elderly persons. J Infect Dis. 2004;189:233–8. 14722887 10.1086/380907

[34] Duncan CB, Walsh EE, Peterson DR, Lee FE-H, Falsey AR. Risk factors for respiratory failure associated with respiratory syncytial virus infection in adults. J Infect Dis. 2009;200:1242–6. 19758094 10.1086/605948 PMC2896971

[35] Thomas S, Ouhtit A, Al Khatib HA, Eid AH, Mathew S, Nasrallah GK, et al. Burden and disease pathogenesis of influenza and other respiratory viruses in diabetic patients. J Infect Public Health. 2022;15:412–24. 35339014 10.1016/j.jiph.2022.03.002

[36] Falsey AR, Hennessey PA, Formica MA, Cox C, Walsh EE. Respiratory syncytial virus infection in Elderly and high-risk adults. N Engl J Med. 2005;352:1749–59. 15858184 10.1056/NEJMoa043951

[37] Lee N, Lui GCY, Wong KT, Li TCM, Tse ECM, Chan JYC, et al. High morbidity and mortality in adults hospitalized for respiratory syncytial virus infections. Clin Infect Dis off Publ Infect Dis Soc Am. 2013;57:1069–77. 10.1093/cid/cit471 23876395

[38] Ackerson B, Tseng HF, Sy LS, Solano Z, Slezak J, Luo Y, et al. Severe morbidity and mortality Associated with respiratory Syncytial Virus Versus Influenza infection in hospitalized older adults. Clin Infect Dis off Publ Infect Dis Soc Am. 2019;69:197–203. 10.1093/cid/ciy991 PMC6603263 30452608

[39] Ackerson B, An J, Sy LS, Solano Z, Slezak J, Tseng H-F. Cost of hospitalization Associated with respiratory syncytial virus infection Versus Influenza infection in hospitalized older adults. J Infect Dis. 2020;222:962–6. 32300806 10.1093/infdis/jiaa183

[40] Thompson WW, Shay DK, Weintraub E, Brammer L, Cox N, Anderson LJ, et al. Mortality associated with influenza and respiratory syncytial virus in the United States. JAMA. 2003;289:179–86. 12517228 10.1001/jama.289.2.179

[41] Binder W, Thorsen J, Borczuk P. RSV in adult ED patients: do emergency providers consider RSV as an admission diagnosis? Am J Emerg Med. 2017;35:1162–5. 28633906 10.1016/j.ajem.2017.06.022

[42] Yu H, Alonso WJ, Feng L, Tan Y, Shu Y, Yang W, et al. Characterization of regional influenza seasonality patterns in China and implications for vaccination strategies: spatio-temporal modeling of surveillance data. PLoS Med. 2013;10: e1001552. 24348203 10.1371/journal.pmed.1001552 PMC3864611

[43] Obando-Pacheco P, Justicia-Grande AJ, Rivero-Calle I, Rodríguez-Tenreiro C, Sly P, Ramilo O, et al. Respiratory Syncytial Virus Seasonality: A Global Overview. J Infect Dis. 2018;217:1356–64. 29390105 10.1093/infdis/jiy056

[44] Uyeki TM, Hui DS, Zambon M, Wentworth DE, Monto AS. Influenza. Lancet Lond Engl. 2022;400:693–706. 10.1016/S0140-6736(22)00982-5 PMC9411419 36030813

[45] Jansen AGSC, Sanders EaM, Hoes AW, van Loon AM, Hak E. Influenza- and respiratory syncytial virus-associated mortality and hospitalisations. Eur Respir J. 2007;30:1158–66. 17715167 10.1183/09031936.00034407

[46] Walsh EE, Peterson DR, Falsey AR. Is clinical recognition of respiratory syncytial virus infection in hospitalized elderly and high-risk adults possible? J Infect Dis. 2007;195:1046–51. 17330796 10.1086/511986

