# Correction: Comparative study on the epidemiological characteristics and hazards of respiratory syncytial virus and influenza virus infections among elderly people

**DOI:** 10.1186/s12879-024-10135-3

**Published:** 2024-11-13

**Authors:** Jiangtao Yu, Na Liu, Yiheng Zhu, Wenyu Wang, Xianquan Fan, Xuan Yuan, Juan Xu, Benfeng Zheng, Lin Luan

**Affiliations:** 1 Department of Epidemiology, School of Public Health, National Vaccine Innovation Platform, Nanjing Medical University, Nanjing, 210000 PR China; 2 Center for Immunization Planning, Chinese Center for Disease Control and Prevention, Beijing, 100050 China; 3 Department of immunization program, Suzhou Center for Disease Control and Prevention, Suzhou, 215000 China; 4 Suzhou Municipal Health Commission, Suzhou, 215002 China

**Correction: BMC Infectious Diseases**

10.1186/s12879-024-10048-1

Following publication of the original article [1], it was flagged to us that the second affiliation of the corresponding author needs to be changed to no. 3, Department of Immunization Program, Suzhou Center for Disease Control and Prevention, Suzhou 215,000, China instead of no.4, Suzhou Municipal Health Commission, Suzhou 215,002, China.

The original article has been corrected.
