# Supplementary material for: Comparative study on the epidemiological characteristics and hazards of respiratory syncytial virus and influenza virus infections among elderly people
Source: BMC Infect Dis. 2024 Oct 9;24:1129. doi: 10.1186/s12879-024-10048-1 (PMC11465698; doi:10.1186/s12879-024-10048-1)
Supplement: Supplementary file 1 — Supplementary Material 1. [file 12879_2024_10048_MOESM1_ESM.pdf]

Questionnaire on Respiratory Infections in Hospitalized Elderly in Suzhou (Patient Medical Record Information Questionnaire)

Investigation of hospital: ______ Date of investigation: ________ Patient Number: __________

1.Basic Information

Name: ______________________

Sex: ______________________

Ethnic: ______________________

Career: ______________________

Telephone call: ______________________

I.D. number: ______________________

Residence address: ______________________

2. Admission Information

Hospitalization number: ______________________

Admission Ward: ______________________

Date of admission: ______________________

Admission diagnosis disease name: ______________________

Admission Diagnosis Disease ICD Code: ______________________

3. Past History

3.1 Does the patient have a chronic underlying condition?

☐No ☐Yes

3.2 The type of chronic underlying disease from which the patient suffers:

☐Cardiovascular disease

☐Diabetes

☐Chronic respiratory diseases

☐Chronic Liver and Kidney Diseases

☐Cancer

☐Other diseases: ______________________

4. Clinical Characteristics, Treatment, and Disease Regression Status

4.1 Since the onset of the disease, has the patient experienced any of the following symptoms or characteristics?

☐Fever, _____℃

☐Cough

☐Runny nose

☐Sputum production

☐Sore throat

☐Wheezing

☐Shortness of breath

☐Dyspnea

☐Other symptoms: ______________________

4.2 Does the patient have any abnormal breath sounds on lung auscultation?

☐No ☐Yes

4.3 Has the patient undergone imaging? If yes, what kind of imaging?

☐No ☐Yes, ______________________

4.4 Has the patient been tested for oxygen saturation? If yes, what was the value of the oxygen saturation?

☐No ☐Yes, ______________________

4.5 Were there any complications during the patient's hospitalization?

☐No ☐Yes

4.6 Types of complications:

☐Pneumonia

☐Otitis media

☐Asthma

☐Respiratory failure

☐Acute respiratory distress syndrome (ARDS)

☐Meningitis

☐Heart failure

☐Hepatic insufficiency and Renal insufficiency

☐Shock

☐Other complications: ______________________

4.7 Treatments:

☐Oxygen therapy

☐Antibiotic therapy

☐Antiviral therapy

☐Corticosteroid hormone therapy

☐Surgery

☐Mechanical ventilation

☐Other treatments: ______________________

4.8 Was the patient admitted to the ICU?

☐No ☐Yes, Date of transfer to ICU: ______________________

Date of transfer out of ICU: ______________________

4.9 Date of discharge: ______________________

4.10 Duration of hospitalization: ______________________

4.11 Discharge outcome:

☐Healing

☐Recover

☐Not healed

☐Death

4.12 Discharge diagnosis

4.12.1 Name of the main diagnosed disease by the western medical doctor discharged from the hospital: ______________________

4.12.2 ICD codes for discharged Western medicine principal diagnosis of disease: ______________________

5. Costs Associated With This Hospitalization

Total costs of hospitalization: ______________________
Comprehensive medical services costs: ______________________
Diagnostic costs: ______________________
Treatment costs: ______________________
Medication costs: ______________________
Consumable costs: ______________________
Other costs: ______________________
