# Supplementary material for: Comparative study on the epidemiological characteristics and hazards of respiratory syncytial virus and influenza virus infections among elderly people
Source: BMC Infect Dis. 2024 Oct 9;24:1129. doi: 10.1186/s12879-024-10048-1 (PMC11465698; doi:10.1186/s12879-024-10048-1)
Supplement: Supplementary file 2 — Supplementary Material 2. [file 12879_2024_10048_MOESM2_ESM.pdf]

Questionnaire on Respiratory Infections in Hospitalized Elderly in Suzhou
(Patient Self-report Questionnaire)

Investigation of hospital: ______ Date of investigation: ________ Patient Number: __________

1.Basic Information

Name: ______________________

Sex: ______________________

Age: ______________________

Are you retired?

☐No ☐Yes

Do you have medical insurance?

☐No ☐Yes

2. Admission Information

2.1 Since the onset of the disease, have you visited a pharmacy to purchase medication?

☐No ☐Yes

2.2 Since the onset of the disease, have you been to any other medical facility or been hospitalized?

☐No ☐Yes

3. Past History

3.1 Vaccination history

Have you gotten a flu shot in the past year?

☐No ☐Yes

Have you received the 23-valent pneumococcal polysaccharide vaccine?

☐No ☐Yes

Have you received the COVID-19 vaccine?

☐No ☐Yes

3.2 Smoking History

☐No ☐Yes, length of time one has smoked______; How many per day? ______

4. Information About This Hospitalization

4.1 How many days are you off work due to this hospitalization?

____

4.2 How much wages you have lost due to this hospitalization?

____

4.3 How many family members have taken time off work due to your current hospitalization?

____

4.4 How many days have your family members taken off work due to your current hospitalization?

____

4.5 How many days did you hire a caregiver for this hospitalization?

____

4.6 How much have you spent on transportation since your current hospitalization?
