# Supplementary material for: Comparative study on the epidemiological characteristics and hazards of respiratory syncytial virus and influenza virus infections among elderly people
Source: BMC Infect Dis. 2024 Oct 9;24:1129. doi: 10.1186/s12879-024-10048-1 (PMC11465698; doi:10.1186/s12879-024-10048-1)
Supplement: Supplementary file 3 — Supplementary Material 3. [file 12879_2024_10048_MOESM3_ESM.pdf]

Informed Consent Form

Dear Participant,

Thank you for participating in the pathogen monitoring and investigation of respiratory infections.

To evaluate the effectiveness of the pneumococcal vaccine as part of our local expanded immunization program and to understand the pathogen spectrum, epidemiological characteristics, and disease burden of acute respiratory infections in hospitalized older adults aged 60 and above, we invite you to participate in a relevant questionnaire survey (which will take approximately 5-6 minutes of your time) and to collect one respiratory specimen for pathogen testing.

This investigation has been reviewed and approved by the Ethics Committee of Suzhou Center for Disease Control and Prevention. Your personal information will be strictly confidential throughout the entire process, and the test results will only be used for the aforementioned evaluation and to provide evidence-based support for local immunization strategies. The results of the pathogen testing of the respiratory specimen will be reported back to the hospital where you were treated within 15 working days.

I have read and understood all the content of the investigation described above, and I agree to participate in the relevant questionnaire survey and provide a respiratory specimen for pathogen testing.

Participant's Signature: _____________

Date: _____ / _____ / _______
